# Editorial: Artificial intelligence-assisted medical imaging solutions for integrating pathology and radiology automated systems

**DOI:** 10.3389/fmed.2024.1447294

**Published:** 2024-06-27

**Authors:** Prabhishek Singh, Manoj Diwakar, Vinayakumar Ravi, Anchit Bijalwan, Tarun Agrawal

**Affiliations:** ^1^School of Computer Science Engineering and Technology, Bennett University, Greater Noida, India; ^2^CSE Department, Graphic Era deemed to be University, Dehradun, Uttarakhand, India; ^3^Graphic Era Hill University, Dehradun, Uttarakhand, India; ^4^Center for Artificial Intelligence, Prince Mohammad Bin Fahd University, Khobar, Saudi Arabia; ^5^School of Computing and Innovative Technologies, British University Vietnam, Hanoi, Vietnam; ^6^Department of CSE and IT, Jaypee Institute of Information Technology, Noida, India

**Keywords:** pathology, radiology, computer-aided diagnosis, explainable AI, generalized models

## 1 Introduction

Pathology and radiology scientists are showing interest in Artificial Intelligence (AI) in the recent time. The advancement of cancer diagnostic methods often incorporates insights from both the domains of pathology and radiography. An integrated diagnostic reporting system that combines textual analysis, representative visuals, and molecular diagnostic data would be advantageous in facilitating informed judgments on the advancement of diagnostic technologies. Artificial intelligence is currently extensively used in a range of diagnostic applications, such as radiography, cancer detection, and infectious disease identification. It is crucial to acknowledge that clinical diagnosis, translational research, and fundamental research are interrelated and might potentially gain advantages from the expanded capabilities of AI. A total of eight publications have been published on this Research Topic. The objective of this Research Topic is to pinpoint the fundamental research challenges related to pathology and radiology in relation to the growing use of AI technology. Furthermore, it is important to have a more profound understanding of the possible uses of AI technology in practical situations.

## 2 Contributions

A total of eight publications have been published on this Research Topic. Waseem Sabir et al. compare the effectiveness of four vision transformers in terms of their capacity to locate anomalies in images derived from computerized tomography (CT) scans and to correctly identify and categorize patients with pulmonary fibrosis. Yuenyong et al. suggested using AI to create a system that can automatically identify centroblast cells by scanning patches from a whole slide image. The AI system is based on the object detection concept, and the only type of object of interest is the centroblast cell. Using 1,669 sample centroblast cell occurrences from whole slide image of five distinct patients, we trained the AI model. The data was divided into training and validation sets of 80% and 20%, respectively. Rauf et al. suggested an 82-layer deep learning architecture based on the residual bottleneck technique has been suggested. An automated computer-aided diagnostic system is needed to identify common maternal fetuses from ultrasound images. The suggested design has incorporated three residual blocks, each with two highway pathways and one skip link. Albalawi et al. investigated the histological images of Oral Squamous Cell Carcinoma and oral epithelium's capacity for discrimination. With a publicly accessible database of 1,224 images taken at different magnifications from 230 patients, a customized deep learning model built on top of EfficientNetB3 was created to distinguish between normal epithelium and OSCC tissues.

Binzagr focuses on the use of SHapley Additive exPlanations (SHAP) technique to understand model predictions. The intermediate layer in this research consisted of a hybrid model of three Convolutional Neural Networks (CNNs)—InceptionV3, InceptionResNetV2, and VGG16—which merged their predictions. This text provides an elucidation of the choice that impacts the model's forecast. Chauhan et al. primarily focuses on the detection of brain tumors to enhance medical study. A Balanced binary Tree CNN (BT-CNN) is presented to enhance the performance of brain tumor classification. The BT-CNN is designed with a binary tree-like structure. The system consists of two separate modules: the convolution module and the depthwise separable convolution group module. Al Moteri et al. presents a sophisticated machine learning model that relies on EfficientNetV2. This model integrates cutting-edge methods in image processing with neural network design, with the goal of enhancing accuracy, speed, and resilience in classification. They used the EfficientNetV2 model, which was specifically adapted for the precise goal of classifying breast cancer images. Imran et al. provide a transformer-based model that can segment histopathological images, distinguishing between inflammation and other types of malignancies. Additionally, the model can detect skin tissues and borders, which are crucial for decision-making purposes. Precise segmentation of these tissue types will ultimately result in precise identification and categorization of non-melanoma skin cancer.

## 3 Conclusion

This editorial published eight research papers that specifically investigated the use of AI-Assisted medical imaging solutions aimed at integrating automated systems in pathology and radiology. The objective was to curate relevant publications within the AI-driven medical and healthcare sector. The findings from this topic highlight a growing trend in the development and research of AI-based medical imaging solutions. To further advance this field, future methodologies could focus on leveraging AI techniques to seamlessly integrate automated systems in both pathology and radiology.

## Author contributions

PS: Writing – review & editing, Writing – original draft. MD: Writing – review & editing, Writing – original draft. VR: Writing – review & editing, Investigation, Conceptualization. AB: Writing – review & editing, Investigation, Conceptualization. TA: Writing – review & editing, Investigation, Conceptualization.

